# Clinical Spectrum of Acute Post-streptococcal Glomerulonephritis in Children: A Kashmir Experience

**DOI:** 10.7759/cureus.63902

**Published:** 2024-07-05

**Authors:** Imtiyaz Ahmad Bhat, Mohd Ashraf, Waseem Shafi Sheikh, Parvez Ahmed, Vaseem Yousuf

**Affiliations:** 1 Pediatrics, Government Medical College Srinagar, Srinagar, IND; 2 Pediatric Nephrology, Government Medical College Srinagar, Srinagar, IND; 3 Pathology, Sher-e-Kashmir University of Agricultural Sciences and Technology of Kashmir (SKUAST-Kashmir), Budgam, IND

**Keywords:** post-streptococcal glomerulonephritis (psgn), pediatric population, acute kidney injury, observational study, acute nephritic syndrome

## Abstract

Background

In the pediatric population, acute post-streptococcal glomerulonephritis (PSGN) is a common glomerular etiology of hematuria and acute hypertension leading to hospitalization. We conducted this study to know the clinical profile and occurrence of acute PSGN in patients presenting with features of acute nephritic syndrome.

Methods

This prospective observational study was conducted on children aged between two and 18 years, presenting with clinical features of acute glomerular nephritis (AGN). After due ethical considerations, all eligible patients were enrolled and underwent detailed clinical assessment, laboratory, and imaging evaluation, followed by protocolized treatment. Relevant data were collected and analyzed to reach valid results.

Results

Out of 60 patients with AGN, PSGN was found in 83.3% of the patients (50/60). The age group under five years was the most commonly involved, with a male/female ratio of 1.6:1. Around half of the studied patients were from the lower middle class, and 40 (80%) were from rural backgrounds. Facial puffiness was the most common clinical presentation, seen in 45 (90%) patients. Hypocomplementemia and proteinuria were seen in all PSGN patients. Pyoderma was the most common preceding infection, seen in 38 (76%) patients, followed by pharyngitis. Acute kidney injury (AKI) was the most common complication, seen in 12 (24%) patients. Complete resolution of the signs and symptoms was seen in 37 (74%) patients at the time of discharge, which increased to 47 (94%) patients at six months post discharge.

Conclusion

PSGN stands to be the most common cause of pediatric AGN. The population under five years of age, with a past history of pyoderma, is more predisposed to PSGN. The potential for the occurrence of AKI and other life-threatening complications is high, for which early diagnosis and institution of proper treatment would be very beneficial.

## Introduction

Diseases involving the renal system are increasingly being encountered in clinical practice. Glomeruli are commonly involved in, during, or after the acute infective process. Acute glomerulonephritis (AGN) classically presents with a sudden onset of hematuria, oliguria, edema, and hypertension in a previously healthy child. Its severity varies from minimal symptoms to severe illnesses, like the presence of anuria, hypertensive encephalopathy, and heart failure [1]. Among the infection-related AGN, post-streptococcal glomerulonephritis (PSGN) is one of the common glomerular causes of hematuria in children. This study was conducted to find out the clinical spectrum and proportion of PSGN among children presenting with features of acute nephritic syndrome/AGN, admitted at a tertiary care pediatric hospital in Kashmir, India.

## Materials and methods

After obtaining institutional ethical clearance from the Ethical Committee of Government Medical College Srinagar (GMCS) (approval no. 180/ETH/GMC/ICM), the present study was conducted in the Department of Pediatrics, GMCS, in Jammu and Kashmir (J&K). This was a prospective observational study conducted from December 2019 to November 2021. All consecutive children aged two to 18 years presenting with acute glomerulonephritis were enrolled in the study. Children suffering from chronic renal disorders, renal stones, and urinary tract infections were excluded from the study.

The diagnosis of acute PSGN was made in the presence of the following criteria [2]: 1) clinical features suggestive of the acute nephritic syndrome; 2) evidence of recent streptococcal infection (e.g., preceding skin infection or pharyngitis and positive anti-streptolysin O titers, anti-DNase B titers, or throat swab positive for group A *Streptococcus*); 3) hypocomplementemia, which means decreased levels of complement 3 (C3) in serum. Serum C3 level normalizes after around eight weeks. Other possible causes like IgA nephropathy, lupus nephritis, and Henoch-Schönlein nephritis need to be ruled out.

Hypertension was defined as systolic blood pressure and/or diastolic blood pressure consistently equal to or exceeding the 95th percentile for an individual’s age, gender, and height on three or more occasions [3]. Hematuria was defined as the presence of five or more red blood cells per high-power field on at least three occasions over a two-three-week period, in a centrifuged urine sample [4]. Acute kidney injury (AKI) was defined as a sudden decrease in renal function within 48 hours, leading to an absolute increase in serum creatinine of more than or equal to 0.3 mg/dl, 50% or more increase in serum creatinine from baseline, or oliguria of less than 0.5ml/kg per hour for more than six hours [5]. Residual renal injury at discharge was defined as abnormal creatinine for age, hypertension, microalbuminuria, or urinary protein creatinine ratio of more than 0.2 [6]. Renal biopsy was performed as per standard criteria [2]. All enrolled patients were managed as per standard protocol, and relevant data were analyzed.

Data was entered in a Microsoft Excel sheet (Microsoft Corporation, USA) and then exported to the data editor of SPSS version 27.0 (IBM Corp., Armonk, NY). Continuous variables were expressed as mean ± SD, and categorical variables were summarized as frequencies and percentages.

## Results

We studied a total of 60 patients fulfilling the criteria of AGN. The most common cause of AGN was found to be PSGN, in 50 out of 60 (83.3%). The commonest age group involved was under five years (46%), and more than 80% of the population were under 10 years of age. Males comprised 62% of the study group, producing a sex ratio of M:F = 1.6:1. It was observed that around half of the patients were from the lower middle class, with 86% of the patients coming from rural backgrounds. Most of our patients developed PSGN during the autumn (42%) and winter (24%) seasons. Common clinical presentations were puffiness of face (90%), swelling of feet (88%), decreased urinary output (76%), and gross hematuria (26%). Proteinuria and hypocomplementemia were seen in all patients of PSGN. Microscopic and gross hematuria were observed in 96% of the patients. Skin infection (pyoderma) was the most common preceding infection seen in 76% of the patients, followed by pharyngitis (24%). AKI was the most common complication among the PSGN patients seen in 24% of cases, followed by hypertensive emergency (16%), cardiac failure (8%), posterior reversible encephalopathy syndrome (4%), and retinopathy (2%). Among the AKI patients, half of them were stage 1, 33.3% were stage 2, and 16.7% were stage 3. As per the pRIFLE criteria, half of the patients had injuries, 25% had a risk of injury, 16.7% had failure, and 8.3% had loss. Other details are mentioned in Table 1.* *Most of the patients with PSGN had normal creatinine values at admission, and 16% had raised creatinine that increased 2.5 times (38%) on 3rd day of admission, as depicted in Table 2. After treatment, and the creatinine levels started to decrease at discharge, at one month follow-up, and six months follow-up with 6% having a residual renal injury. All the patients of PSGN had proteinuria on admission. Most of them had non-nephrotic proteinuria (78%), while 11 (22%) had nephrotic-range proteinuria. It was observed that 48 (96%) patients had nil proteins in urine at one month follow-up, as mentioned in Table 3.

**Table 1:** Clinical parameters of the PSGN patients PRES: posterior reversible encephalopathy syndrome, AKI: acute kidney injury, AKIN: acute kidney injury network, pRIFLE: pediatric risk, injury, failure, loss, end-stage renal disease, PSGN: post-streptococcal glomerulonephritis

Characteristics		Number	Percentage
Age in years	Mean	6.5 ± 3.19	
	<5	23	46
	5-10	18	36
	>10	9	18
Gender	Male	31	62
	Female	19	38
Preceding infection	Pharyngitis	12	24
	Pyoderma	38	76
Season	Spring	8	16
	Summer	9	18
	Autumn	21	42
	Winter	12	24
Blood pressure	Normal	4	8
	Hypertension	38	76
	Hypertensive emergency	8	16
Complications	Acute kidney injury	12	24
	Hypertensive emergency	8	16
	Congestive heart failure	4	8
	PRES	2	4
	Hypertensive retinopathy	1	2
Stage of AKI (as per AKIN classification, n=12)	Stage 1	6	50.0
	Stage 2	4	33.3
	Stage 3	2	16.7
AKI (as per pRIFLE criteria, n=12)	Risk	3	25.0
	Injury	6	50.0
	Failure	2	16.7
	Loss	1	8.3
	End stage	0	0.0
	Requirement for dialysis	1	2
Duration of hospital stay (Days)		6.3 ± 1.79	
Outcome at discharge	Complete resolution of symptoms	37	74
	Partial resolution of symptoms	13	26

**Table 2:** Laboratory parameters of the PSGN patients Values are depicted as mean ± standard deviation and n (%). PSGN: post-streptococcal glomerulonephritis

Lab parameter	Characteristics	Measured values
Serum creatinine level (mg/dl)	On day 1 of admission	0.58 ± 0.208
	Maximum level reached	0.86 ± 0.651
	At discharge	0.61 ± 0.209
	After six months	0.46 ± 0.147
Blood urea nitrogen (mg/dl)		30.7 ± 8.31
Urine protein-to-creatinine ratio	<0.2 (Normal)	0 (0)
	0.2-2.0 (Non-nephrotic proteinuria)	39 (78)
	>2.0 (Nephrotic proteinuria)	11 (22)
Positive ASO titre (>200 units/ml)		11 (22)
Serum anti-DNase B (units/ml)		225.3 ± 108.8
Serum C3 (g/L) at admission		0.26 ± 0.114

**Table 3:** Parameters of PSGN patients on follow-up All values are depicted as n (%). PSGN: post-streptococcal glomerulonephritis

Parameter	Characteristics	At one month	At six months
Blood pressure	<95th percentile	48 (96)	49 (98)
	≥95th percentile	2 (4)	1 (2)
Urine protein-to-creatinine ratio	<0.2 (Normal)	48 (96)	49 (98)
	0.2-2.0 (Non-nephrotic proteinuria)	2 (4)	1 (2)
	>2.0 (Nephrotic proteinuria)	0 (0)	0 (0)
Abnormal creatine levels for age		8 (16)	3 (6)

In all patients with PSGN, C3 levels were below normal, while C4 levels were within the normal range. While the anti-streptolysin O (ASO) titers were elevated (>200) in 11 (22%) patients, the anti-DNase B levels were on the higher side (>170) in 38 (76%) patients. ESR and CRP were also increased in more than 50% of the study population. Only 10% of PSGN patients needed renal biopsy and common indications were nephrotic range proteinuria and systemic illness with proteinuria. Most of the patients (74%) had complete resolution of symptoms at discharge, while 26% had partial resolution. While 94% of the patients had complete resolution of symptoms at six months follow-up, only 6% had partial resolution of symptoms. There was no mortality among the PSGN patients included in our study.

## Discussion

All hospitalized children fulfilling the diagnostic criteria of acute glomerulonephritis were managed using a standardized protocol. Out of 60 AGN patients, the proportion of PSGN was 50 (83%), consistent with the existing literature that states that the commonest cause of acute glomerulopathy among children is PSGN [7]. Out of 50 PSGN children, there were 31 (62%) males and 21 (38%) females, i.e., a male-female ratio of 1.6:1, which is similar to the observations made by earlier studies [8]. However, one study has reported that 56% of PSGN patients were females between the ages of two months to 15 years [9]. Based on the observations of the present study, PSGN is more frequent in children aged two to 14 years, with a mean age of 6.5 ± 3.19 years. This is similar to the observations made in other studies around the world [10,11,12]. Most of our patients were from the middle and lower class with a rural background. This is similar to the observations made by Cruz et al., who reported that out of 124 children studied, the majority of patients belonged to a lower socioeconomic group, and 54% of the families had incomes below the poverty line [13]. The involvement of these social strata could be due to poor vaccination administration rates, overcrowding, and large families, besides poor nutrition. More cases of PSGN were observed during autumn (42%) and winter (24%) seasons, which could be due to more rainfall during these seasons and thereby more skin infections. This conforms with the study done by Ibadin et al. [9]. Most of our PSGN patients were related to post-pyoderma sequelae, while post-pharyngitis-associated PSGN were lower in number, which is similar to the earlier studies [5,9].

Pertaining to the clinical features of our patients, we observed that patients presented with puffiness of the face (90%), feet swelling (88%), and decreased urine output (88%). These findings are very much in congruence with the studies done around the world [7,13,14]. We observed proteinuria and hypocomplementemia in all patients (100%), while hematuria was observed in 48 (96%) patients. We found elevated levels of ASO titers in 11 (22%) and anti-DNase B in 38 (76%) patients, which were the objective evidence of recent streptococcal infection prior to PSGN. These findings are similar to the observations made by Puri et al. [15] and Manhas et al. [16]. Regarding complications, we observed AKI in 12 (24%), hypertensive emergency in eight (16%), cardiac failure in four (8%), and PRES in two (4%) patients, while retinopathy was detected in 1 (2%) patient, which conforms with the reports of Gunaskeran et al. [17] who found AKI in 20.8% of PSGN patients. The incidence of AKI was 24% in our study, with 50% of them having stage 1 AKI. On applying pRIFLE, most of the patients fell in the injury category.

The creatinine trend in the present study showed a sharp rise within the first three days, which was followed by a gradual decline. At the time of admission, eight (16%) patients had creatinine levels above their age and gender-specific range, which jumped to 19 (38%) patients on the third day. It gradually resolved, and only three (6%) patients had persistent deranged creatinine levels after a gap of six months from the admission day. However, proteinuria was detected in 11 (22%) patients at the time of admission, which declined during the hospital stay, and at the end of one month, only two (4%) patients had proteinuria, while by the end of six months, only one (2%) patient was affected by non-nephrotic proteinuria. All eight (16%) hypertensive encephalopathy patients were managed in the PICU and were successfully discharged. We observed that most of the patients who had hypertension resolved within 7-12 days, which is in line with the existing medical literature [18]. We observed an almost complete resolution of clinical and biochemical parameters by the end of six months, while only three (6%) patients had partial recovery.

Our study had some limitations. Our study was single-center hospital-based and not representative of the general population. Only symptomatic patients were included, which is an underestimation of the true burden of the disease. Due to a lack of long-term follow-up, the sequelae of PSGN cannot be commented upon.

## Conclusions

School-age children with a recent history of skin lesions from rural areas of Kashmir Valley are prone to develop PSGN. Children who commonly present with hypertension, gross hematuria, edema, and/or oliguria can develop AKI, hypertensive emergency, and cardiac failure. Prompt diagnosis and adequate treatment yields better outcomes.
